# Increasing the distance between two monomers of topoisomerase IIβ under the action of antitumor agent 4*β*-sulfur-(benzimidazole) 4′-demethylepipodophyllotoxin

**DOI:** 10.1038/s41598-018-33366-2

**Published:** 2018-10-08

**Authors:** Lin-Yang Sun, Li-Wen Zhu, Ya-Jie Tang

**Affiliations:** Hubei Key Laboratory of Industrial Microbiology, Hubei Provincial Cooperative Innovation Center of Industrial Fermentation, Key Laboratory of Fermentation Engineering (Ministry of Education), Hubei University of Technology, Wuhan, 430068 China

## Abstract

Topoisomerases II (Top2s) are a group of essential enzymes involved in replication, transcription, chromosome condensation, and segregation via altering DNA topology. The mechanism of the Top2s poisons such as etoposide (VP-16) was reported as stabilizing the Top2-DNA complex and engendering permanent DNA breakage. As the structurally similar compound of VP-16, a novel 4*β*-sulfur-substituted 4′-demethylepipodophyllotoxin (DMEP) derivative (compound C-Bi) with superior antitumor activity was developed in our previous study. To understand the structural basis of the compound action, the crystal structure (2.54 Å) of human Top2 β-isoform (hTop2β) cleavage complexes stabilized by compound C-Bi was determined. However, compound C-Bi was not visible in the crystal structure. Through the comparison of the structures of hTop2β-DNA-etoposide ternary complex and hTop2β-DNA binary complex, it could be observed that the distance between drug-binding sites Arg503 of the two monomers was 26.62 Å in hTop2β-DNA-etoposide ternary complex and 34.54 Å in hTop2β-DNA binary complex, respectively. Significant twist were observed in the DNA chains of binary complex. It suggested that compound C-Bi played antitumor roles through increasing spacing of hTop2β monomers. The changes in hTop2β structure further caused double changes in the torsional direction and migration distance of the DNA chains, resulting in impeding religation of DNA.

## Introduction

Topoisomerases II (Top2s) are a group of essential nuclear enzymes that alter the topological structure of DNA through the transient cleavage, strand passage and subsequent re-ligation of double-stranded DNA^1–4^. Top2s in mammalian cells can be divided into two subtypes which are type IIα (Top2α) and IIβ (Top2β)^5^. Top2α plays roles in proliferating cells and assists in replication, chromosome condensation and segregation^6,7^. And Top2β is associated with DNA repair and transcription^2,8^. Especially, site-specific cleavage of DNA by human topoisomerases IIβ (hTop2β) has been reported to be necessary to activate the transcription of some genes^9^.

Beyond the vital cellular functions, Top2s are considered as an important target protein for some of the most active drugs for the treatment of human cancers^10–12^. Top2s-targeting drugs, such as etoposide (VP-16), are among the most effective and widely used anticancer drugs in cancer chemotherapy^13–15^. These drugs are referred to as Top2s poisons, because they convert Top2s into a physiological toxin that creates DNA double-strand breaks by increasing the steady-state levels of DNA cleavage complex^16,17^. Top2s-mediated DNA damage can further activate a series of intracellular signal response, and ultimately lead to tumor cells death^18,19^. To understand the structural basis of Top2s-targeting drug, especially Top2β-targeting, the high-resolution crystal structure of a ternary VP-16-hTop2β catalytic core-DNA complex has recently been determined, which revealed important information on the action mode of VP-16^20,21^. As the structurally similar compounds of VP-16, a 4′-demethylepipodophyllotoxin (DMEP) derivative modified by a carbon-sulfur bond (i.e., 4β-S-(benzimidazole)-DMEP) (compound C-Bi) was developed in our previous work, which exhibited superior antitumor activity, the inhibition activity of target proteins Top2s, and apoptosis induction to that of VP-16^22^. However, the molecular mechanism underlying the difference is not well understood. Herein, the crystal structure of the catalytic core of hTop2β in complex with DNA and DMEP derivative was determined. The results can provide useful information for better understanding of the action mechanisms of Top2s-targeting antitumor agents.

## Results

### Detecting hTop2β-DNA covalent complexes using the ICE (***in vivo*** complex of enzyme) bioassay

**Figure 1:**
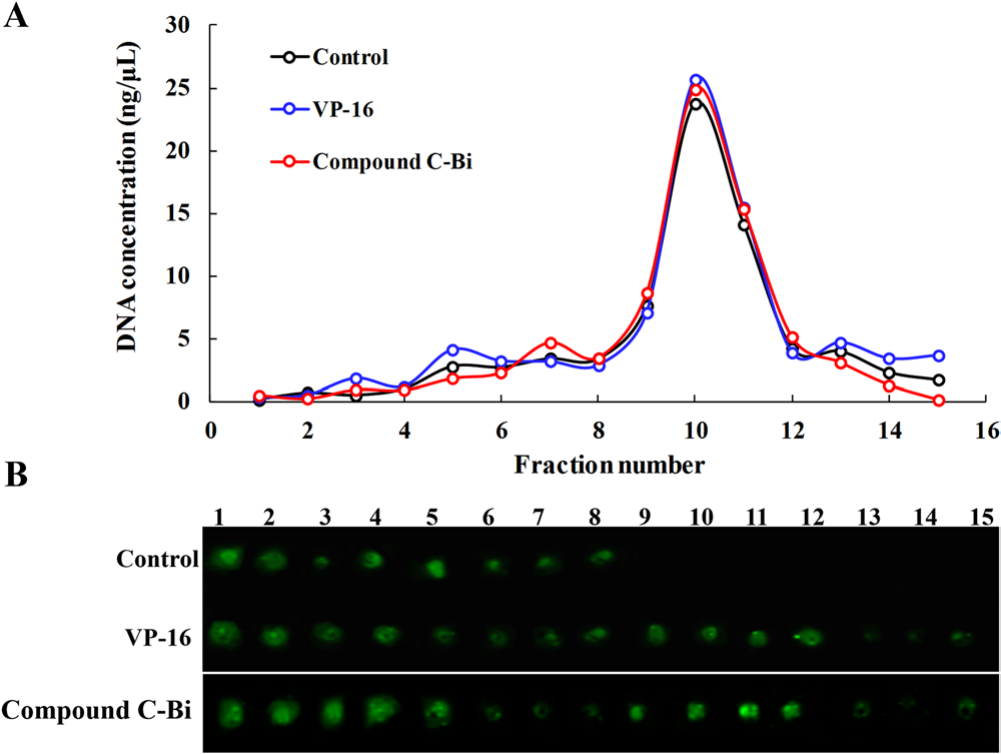
Detection of DMEP derivatives-induced hTop2β-DNA covalent complexes in HeLa cells. HeLa cells (1 × 10^7^) were treated with 100 μM drugs for 30 min at 37 °C. (**A**) DNA profiles of untreated and 100 μM VP-16 or compound C-Bi treated cells. (**B**) Immunoblot data of gradient fractions from untreated and 100 μM VP-16 or compound C-Bi treated cells. The spots of compound C-Bi came from another nitrocellulose membrane (For complete blots we refer to Supplementary Fig. S1). The two membranes were operated and photographed under the same condition without overexposure or exposure adjustment.

The ICE bioassay detected the hTop2β-DNA covalent complex formation *in vivo* with HeLa cells. Exponentially growing cultures were either treated with drugs (100 μM) or without drug. And then processed for covalent complexes. In the CsCl gradients (Fig. 1A), DNA mainly distributed at the expected density in fractions 9–12. Then, immunoblotting of the gradient fractions using an appropriate antibody measures hTop2β. As shown in Fig. 1B, free proteins were in the top fractions 1–8. No stabilized hTop2β-DNA intermediates were detected in the absence of drug, indicating a lack of cross-reactivity between hTop2β and DNA. In the presence of VP-16 or compound C-Bi, hTop2β was detected in the DNA peak fractions. It clearly suggested that the formation of hTop2β-DNA covalent complexes could be promoted under the action of VP-16 or compound C-Bi.

### DNA cleavage assay

**Figure 2:**
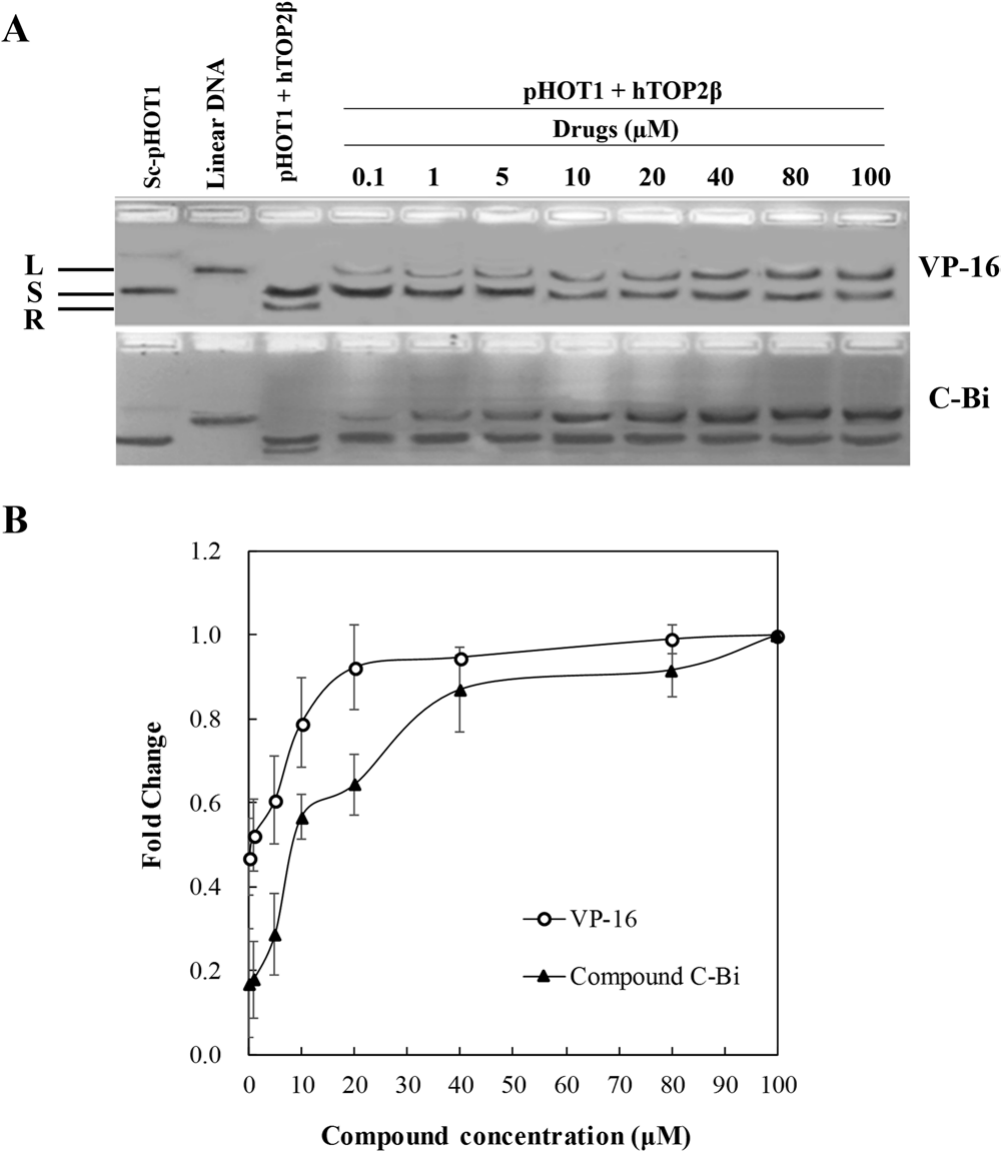
Concentration dependent effect of compound C-Bi and VP-16 on the hTop2β-mediated cleavage of supercoiled pHOT1 plasmid DNA to produce linear DNA. (**A**) The linear DNA detection of different reaction systems. The concentration series were 0.1, 1, 5, 10, 20, 40, 80, 100 μM for compound C-Bi and VP-16. L, linear DNA; S, supercoiled DNA; R, relaxed DNA. (**B**) The gray scale value of the linear DNA amount. Data plotted are the mean ± SD of three separate experiments.

To determine whether the DNA cleavage induced by compound C-Bi is associated with the interruption of topoisomearse II activity, a cell-free DNA cleavage assay using an enzyme-mediated negatively supercoiled pHOT1 plasmid DNA was applied. A representative gel image of the relaxation assay for determining the catalytic inhibition on hTop2β was presented in Fig. 2A. Topoisomerase IIβ poisons such as VP-16 were known to stabilize the topoisomerase IIβ-DNA complex that lead to DNA breaks and generate linear DNA^23,24^. In agarose gel, linear DNA (L) was difficult to enter and appeared at the top; whereas the relaxed DNA (R), and supercoiled DNA (S) move easily into the gel. As shown in Fig. 2A, compound C-Bi stabilized hTop2β cleavage complexes and exhibited the formation of linear DNA. This result shows that compound C-Bi acted as hTop2β poison. To more clearly describe the concentration-dependent effect on linear DNA, a gray-scale analysis was performed (Fig. 2B). It was observed that the DNA cleavage increased with an increase in the concentration of VP-16 and compound C-Bi. It indicated that VP-16 and compound C-Bi stabilized or trapped the Top2-cleaved DNA complex in a concentration-dependent manner.

### Binding of DMEP derivatives to hTop2β-DNA complex

**Figure 3:**
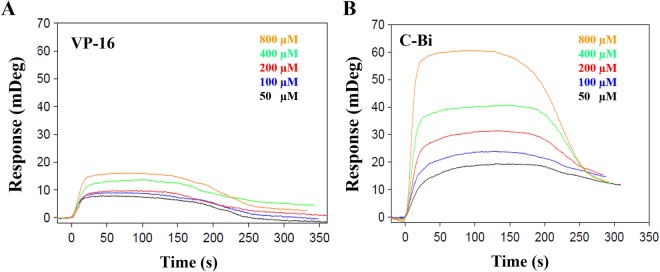
The interaction between compound C-Bi and VP-16 with immobilized hTop2β-DNA complex was measured by surface plasmon resonance (SPR). (**A**) VP-16; (**B**) Compound C-Bi. In each case, the signal is proportional to the compounds concentration (For complete kinetics parameters we refer to Supplementary Table S1). The largest signal corresponds to the highest compound concentration and vice versa.

To further investigate the binding affinity of compound C-Bi and VP-16 to hTop2β-DNA complex, surface plasmon resonance (SPR) was employed. As shown in Fig. 3, the response unit increased in a concentration-dependent manner. The equilibrium dissociation constant (*K*_*D*_) of compound C-Bi (45.9 ± 3.6 μM) were approximately 2.5 folds lower than that of VP-16 (113.6 ± 6.4 μM). These data indicated DMEP derivative exhibited higher affinity for hTop2β-DNA complex than VP-16.

### Structure basis of the complex

**Figure 4:**
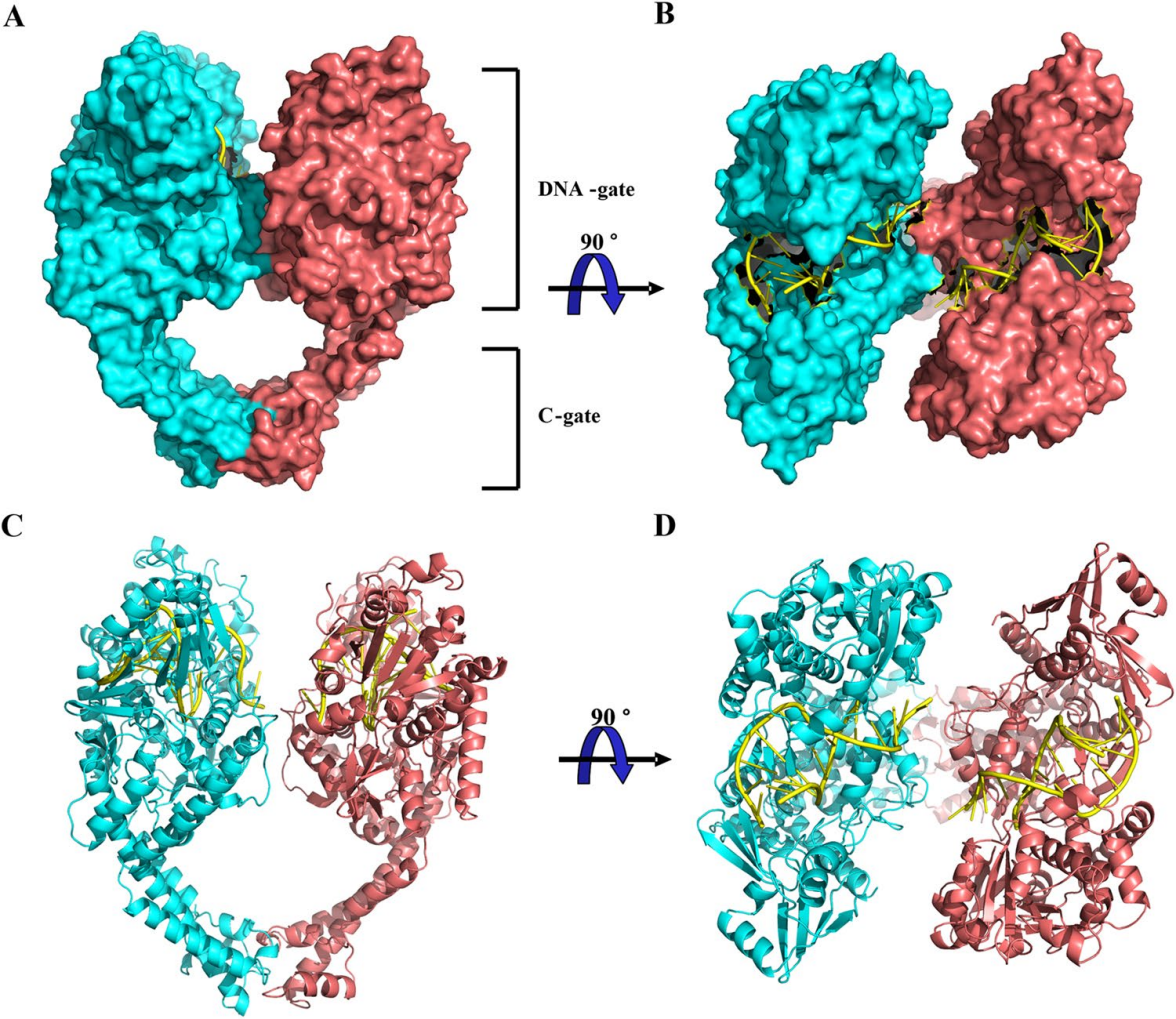
Structure of hTop2β-DNA complex. (**A**,**B**) Orthogonal views of the hTop2β-DNA complex from front view (**A**) and top view (**B**). (**C**,**D**) The cartoon representation of the hTop2β-DNA complex from front view (**C**) and top view (**D**). The dimeric hTop2β protein was shown as a surface representation and colored according to polypeptide chain. DNA was shown in yellow.

To provide insights into the interactions of compound C-Bi with hTop2β at the molecular level, we initially sought to investigate the molecular mechanism of action of compound C-Bi. Using the well-established system for hTop2β crystal growth^20^, the crystal of hTop2β cleavage complex containing two monomers of hTop2β and cleaved DNA chain was obtained under the action of compound C-Bi with the resolution of 2.54 Å (Table 1, Fig. 4A,B). The DNA was embedded in the hTop2β (Fig. 4C,D). All DNA base pairs were visible in the electron density maps (Supplementary Fig. S2). However, no molecule of compound C-Bi was observed in the stabilized hTop2β cleavage complex structures.

**Table 1:** Summary of crystallographic analysis.

Structure	5ZAD
**Data Collection**	
Wavelength	0.97845
Space group	P32
**Cell dimensions**	
*a*, *b*, *c* (Å)	95.006, 95.006, 230.289
*α*, *β*, *γ* (^o^)	90, 90, 120
Resolution range (Å)	47.50–2.20
No. of unique reflections	118178
Completeness (%)	99.5
*Rsym*	0.083
**Refinement**	
Resolution range (Å)	28.06–2.54
Rwork/Rfree (%)	18.0/22.7
No. of reflection	76508
Bond lengths (Å)	0.009
Bond angles (^o^)	1.25
**Ramachandran statistics**	
Favored regions (%)	94.54
Outliers (%)	0.21

**Figure 5:**
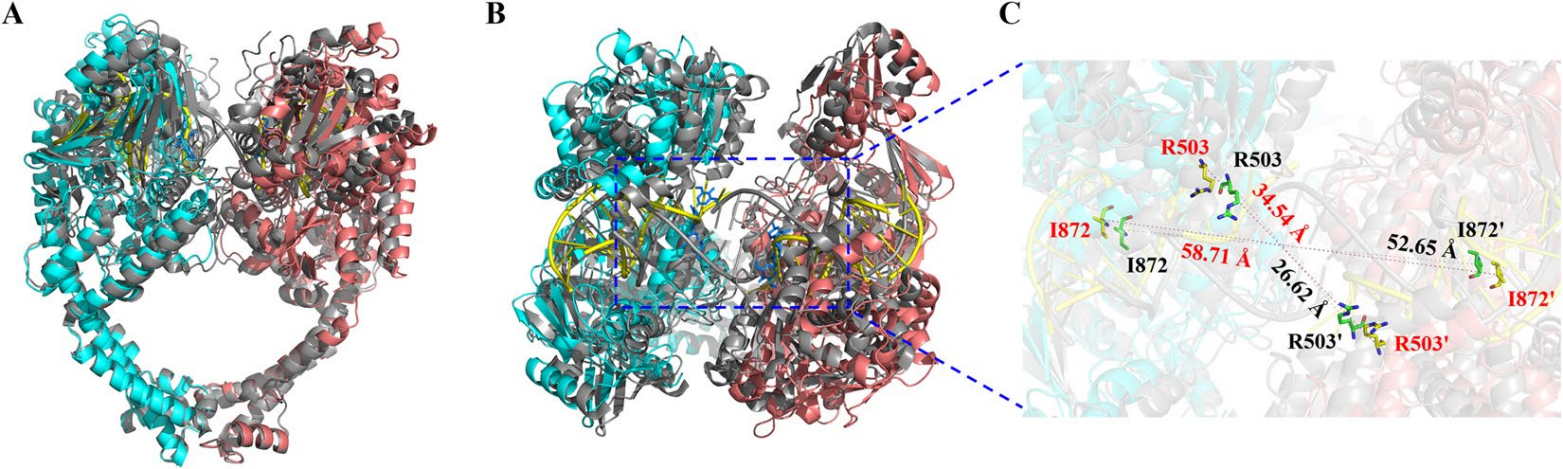
Comparison the crystal structures of hTop2β-DNA complex (PDB: 5ZAD and 3QX3). (**A**,**B**) The superimposition of the hTop2β-DNA under the action of compound C-Bi (PDB ID 5ZAD: cyan and red) and hTop2β-DNA-VP-16 (PDB ID 3QX3: grey) structures from front view (**A**) and top view (**B**). VP-16 was colored blue. (**C**) Selected residues from the enclosed region were shown in an enlarged view to illustrate the change of distance between two monomers. The distances between residues involved in drug-binding (R503) and DNA-intercalating (I872) were indicated as dashed lines. Residues with labels from different structures were colored differently (3QX3 in green and black and 5ZAD in yellow and red). Oxygen and nitrogen atoms were colored red and blue, respectively. Labels belonging to the second monomer were flagged by a prime.

In order to understand the mechanism of compound C-Bi, the structures of hTop2β-DNA binary complex and hTop2β-DNA-etoposide ternary complex were compared. The RMSD value for this alignment was 3.604 Å (1304 to 1304 atoms). The structure of the hTop2β-DNA binary complex revealed a looser assembly pattern than in hTop2β-DNA-etoposide ternary complex (Fig. 5A,B) and other DNA-bound structures (Supplementary Table S2). However, the changes of structure were mainly located in the DNA-gate, and there was no obvious change in the C-gate. The distance between the main DNA-contacting domains of the two monomers was significantly increased under the action of compound C-Bi (Fig. 5C). The distance between drug-binding sites Arg503 of the two monomers was 26.62 Å in hTop2β-DNA-etoposide ternary complex and 34.54 Å in hTop2β-DNA binary complex, respectively. The distance between DNA-intercalating sites Ile872 of the two monomers was 52.65 Å in the ternary complex, and 58.71 Å in the binary complex.

**Figure 6:**
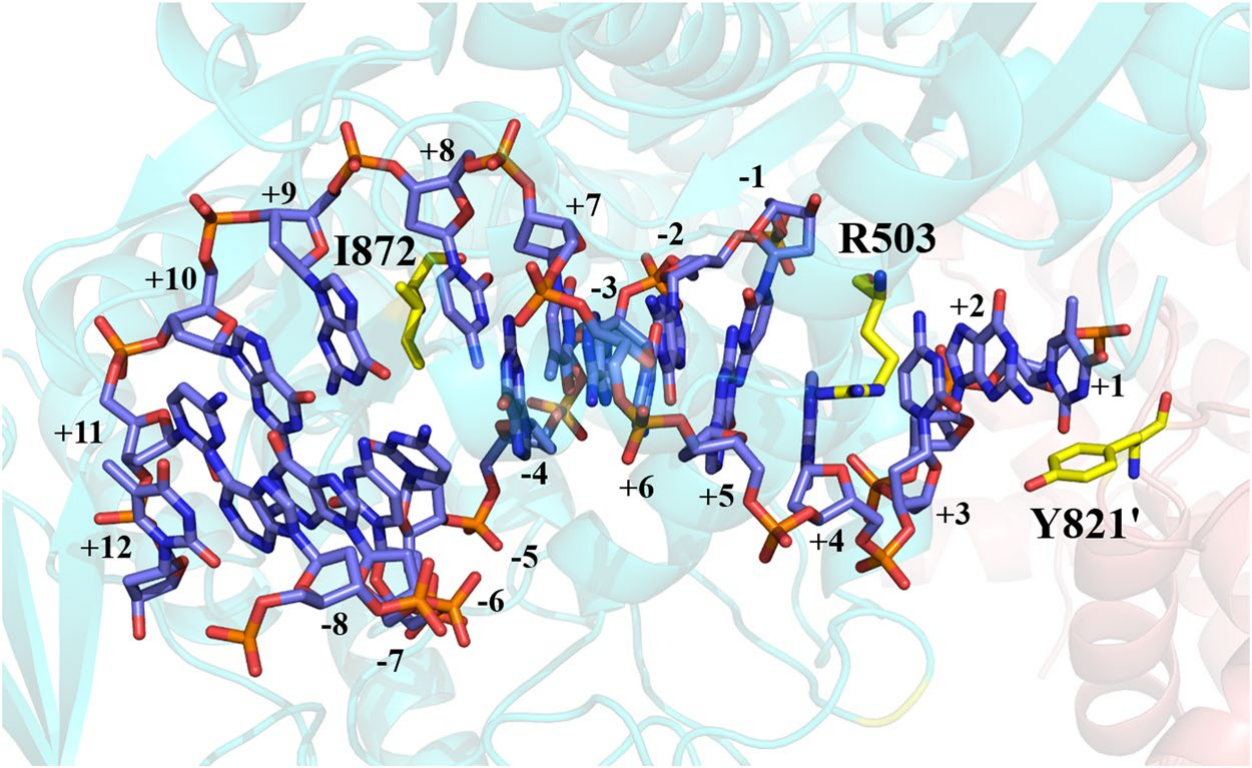
Detailed views of the DNA cleavage site of the hTop2β-DNA binary complex. Positive and negative numbers designated nucleotides downstream and upstream of the scissile phosphate. DNA was shown as blue sticks. Key residues were shown as yellow sticks. Oxygen and nitrogen atoms were colored red and blue, respectively. The two hTop2β monomers were colored differently. Labels belonging to the second monomer were flagged by a prime.

Arg503 is a major drug-contacting residue^20,21^. In hTop2β-DNA-etoposide ternary complex, for interacting with VP-16, the +5 guanine base could form a hydrogen bond with the main-chain carbonyl of Arg503^20^. In this work, perhaps because of the loose assembly of monomers, the distance between Arg503 and +5 guanine base was increased, and no hydrogen bond was observed (Fig. 6). Similarly, the phosphotyrosyl linkage that should existed between +1 nucleotide and active site residue Tyr821’ was weaken. DNA-intercalating isoleucine (Ile872) still bound between the +8 and +9 bases.

**Figure 7:**
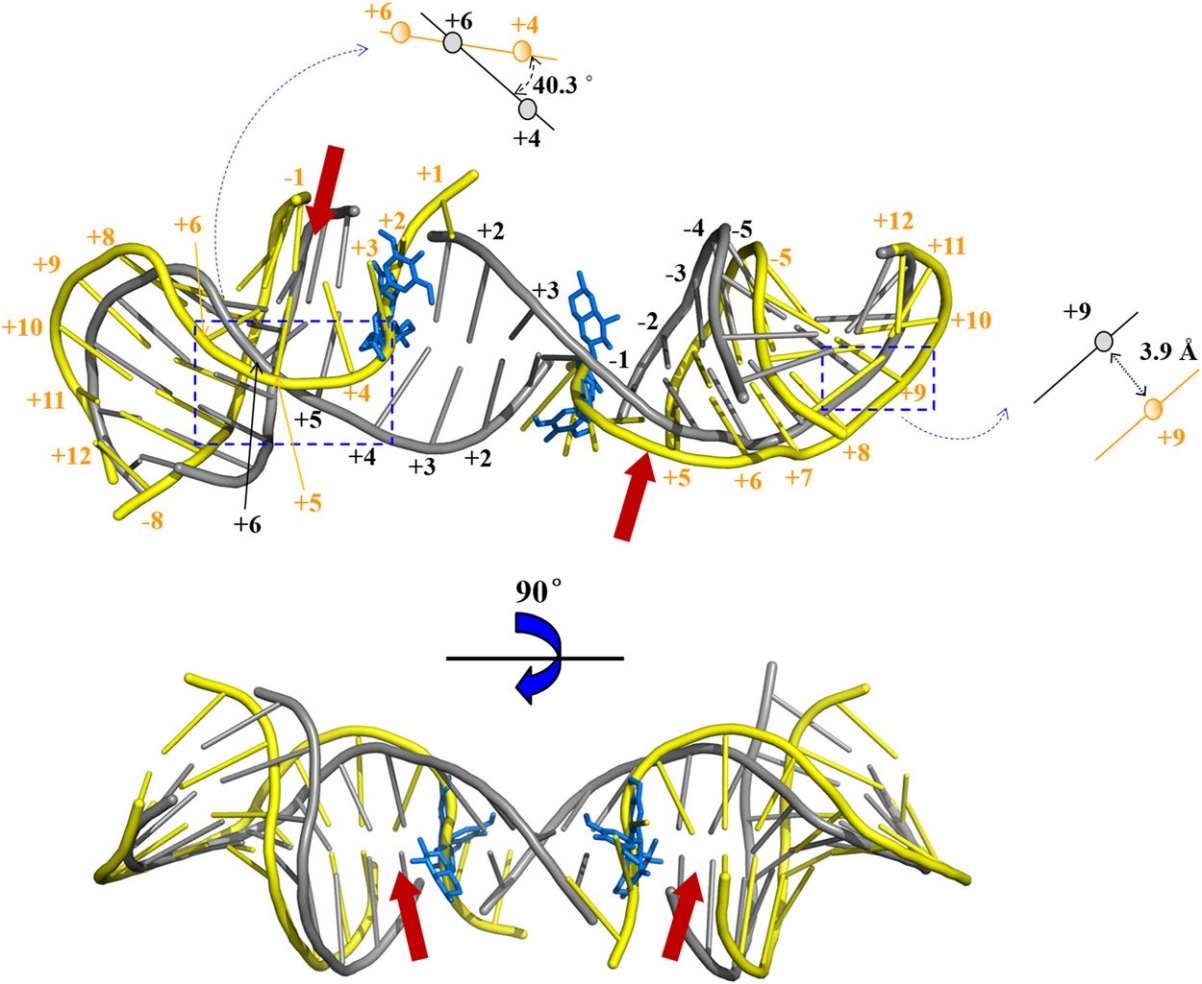
The superimposition of DNA molecules from hTop2β-DNA binary cleavage complex (PDB ID 5ZAD: yellow) and hTop2β-DNA-etoposide ternary cleavage complex (PDB ID 3QX3: grey). VP-16 was shown in blue stick representation. The red arrow represented the conjectural binding site of compound C-Bi. The comparison of the changes in configuration by calculating the angle and the distance for the structure of the enclosed region were shown from close-up view. The angle calculation was based on the overlapping point of the top view projection of DNA chains. This overlapping point was approximately +6 base of hTop2β-DNA-etoposide ternary cleavage complex. The labels of base were colored orange (PDB ID 5ZAD) and black (PDB ID 3QX3), respectively.

To compare the differences between DNA molecules, the superimposition of DNA molecules from hTop2β-DNA binary cleavage complex and hTop2β-DNA-etoposide ternary cleavage complex were shown. The insertion of VP-16 abolished the stacking interaction between the +1/+4 and −1/+5 base pairs. Thus, a drug-binding site for compound C-Bi between the same base pairs in hTop2β-DNA binary complex was assumed. More obvious twist have been observed in the DNA chains of binary cleavage complex (Fig. 7). Compared with that of VP-16, the DNA chain configuration was changed by approximately 40.3° of the angle and 3.9 Å of distance under the action of compound C-Bi.

## Discussion

VP-16 is one the most effective anticancer drugs in clinical use, the mechanism of which was reported as stabilized the hTop2β-DNA complex by bonding at the two scissile bonds of a hTop2β-DNA cleavage complex. In our previous work, a novel 4β-sulfur-substituted DMEP derivative was developed, which exhibited superior antitumor activity to that of VP-16^22^. As the structurally similar compound of VP-16, the antitumor mechanism of compound C-Bi remains largely unknown.

Through ICE assays, DNA cleavage assay and kinetic analysis, we have identified compound C-Bi could bind to hTop2β, and stabilize the formation of ternary complex. To further understand the mechanism, the crystal structure (2.54 Å) of hTop2β cleavage complexes stabilized by compound C-Bi was determined. However, no molecule of compound C-Bi was observed in the hTop2β cleavage complex structures. In our study, the stable hTop2β-DNA complex cannot be formed without the action of DMEP derivative (data not shown). In the previous work, the hTop2β-DNA binary complex can only be obtained through soaking out of VP-16 from the hTop2β^core^-DNA-etoposide ternary complex^21^. By comparison of the structure of hTop2β^core^-DNA-VP-16 ternary complex (PDB: 3QX3) and hTop2β-DNA binary complex (PDB: 4J3N), the difference was only the absence of VP-16. It indicated that although there was no compound C-Bi in the complex structure, the crystal structure could still provide information for explaining the mechanism of compound C-Bi action.

To assess the possible mechanism of compound C-Bi, we compared the structure mode of hTop2β^core^-DNA binary complex (PDB: 5ZAD) and hTop2^core^-DNA-etoposide ternary complex (PDB: 3QX3). In the crystal structure of hTop2β^core^-DNA complex under the action of compound C-Bi, with the two monomers moving away from each other, the distances between the drug-contacting sites (Arg503 and Arg503′) and between the two DNA-intercalating isoleucines (Ile872 and Ile872′) are longer than those observed in VP-16-stabilized structures (Fig. 5, Supplementary Table S2). Increased spacing may suggest an intermediate state between a closed state^25^ and the open conformation^26^. It indicated the action of compound C-Bi needed a looser packing between the two monomers. It was speculated that the amplitude of hTop2β motions increased under the effect of compound C-Bi. Therefore, the intermediate states of the movement of two monomers could be easily captured.

The change of cleavage DNA was also observed with more significant twist of DNA chains (Fig. 7). Compared with VP-16, the action of compound C-Bi led to further migration of the two DNA chains. That may be caused by the increased distance between the two monomers. The interaction between the residues and the base was weakened or eliminated by the migration of the monomers. DNA chains were more significantly twisted, and some bases could not be paired with each other (+1/+4). Lack of base-pairing and torsion of the DNA chains leads to an inability to re-ligate the DNA break, thus preventing repair of the DNA damage. When Top2s play roles in replication, transcription, chromosome condensation, and segregation, Top2 alters DNA topology via the formation of a Top2-DNA cleavage complex^26^. Top2 poisons (e.g. VP-16) inhibit the religation of cleaved DNA resulting in DNA damage^27^. Accumulation of Top2-mediated DNA damage leads to cell death. It suggested that compound C-Bi played antitumor roles through increasing spacing of hTop2β monomers. The changes in hTop2β structure further caused double changes in the torsional direction and migration distance of the DNA chains, resulting in impeding religation of DNA.

In summary, to understand the structural basis of compound C-Bi action, we determined the interaction of compound C-Bi with hTop2β and DNA using X-ray crystallography. By comparing the structures of the complexes, the distance between the two monomers of hTop2β was increased, and significant twist of DNA chains was found under the action of compound C-Bi. It suggested that compound C-Bi played antitumor roles through increasing the change of hTop2β monomers structure and structural changes will further generated DNA damage. The structure information reported here advanced the understanding of the inhibitory mechanisms of Top2-targeting anticancer compounds.

## Methods

### Construction and expression of hTop2β^core^

The hTop2β^core^-PET51b plasmid was kindly provided by Dr. Nei-Li Chan at National Taiwan University^20^. The plasmid was transformed into *Escherichia coli* BL21 (DE3) pLysS cells. For expression, the transformed strain was grown in LB medium at 37 °C to an OD_600_ = 1.0. Isopropyl-β-D-thiogalactopyranoside (IPTG) (Biosharp, Seoul, Korea) was added to a final concentration of 0.3 mM, and protein expression was induced at 20 °C for 16 h.

### hTop2β^core^ purification

The cells were centrifuged at 5000 rpm for 15 min (4 °C), and the pellets were resuspended in lysis buffer containing 50 mM NaPi (pH 7.4), 10% glycerol, 500 mM NaCl, 5 mM β-mercaptoethanol, 0.5 mM phenylmethanesul fonylfluoride, and 10 mM imidazole^20^. And then the cells were disrupted using a high pressure cell crusher (AH-1500, ATS, Canada) at 4 °C. Unbroken cells were removed by centrifugation at 15,000 rpm for 30 min. The hTop2β^core^ protein was isolated from the cell lysate supernatant using Ni-NTA column (Clontech, USA) and eluted with elution buffer containing 250 mM imidazole without NaCl. The resulting protein was loaded onto a HiPrep 16/10 Heparin FF column (GE Healthcare). The protein was eluted in a linear gradient over 10 column volumes with buffer A (30 mM Tris-HCl pH 7.5, 15 mM NaCl, 2 mM β-mercaptoethanol, and 1 mM EDTA) and buffer B (buffer A containing 1 M NaCl). The eluted fractions were pooled and purified on a gel filtration column (Superdex 200, GE Healthcare) in buffer C (buffer A containing 70 mM NaCl). The hTop2β^core^ protein (with molecular weight about 180 kDa) was collected and concentrated to 6.5 mg/ml for crystallization.

### DNA substrate for crystallography

The DNA sequence 5′-AGCCGAGCTGCAGCTCGGCT-3′ of the double-stranded DNA substrate was prepared as previously described^20^. Synthesis and purification of DNA were performed by a commercial company (GenScript, Nanjing, China). The oligonucleotides were dissolved in buffer containing 30 mM Tris-HCl (pH 7.5), 70 mM NaCl, 2 mM β-mercaptoethanol, and 1 mM EDTA) and annealed at 55 °C to generate double-stranded DNA for crystallization.

### ICE assay

The ICE bioassay was performed as previously described^28,29^. HeLa cells were cultured in 25 cm cell culture dishes at 37 °C, and a single Petri dish was used per treatment (90% confluent, approx 1 × 10^7^ cells). The medium was removed and replaced with serum-free medium (1 mL) containing topoisomerase inhibitors (e.g., 100 μM etoposide or compound C-Bi, final concentration) and cells were incubated at 37 °C for 60 min. The negative control containing no drug (solvent only) was prepared in parallel. Then the drug containing medium was removed, and cells were lysed by the 1% sarkosyl equilibrated to 37 °C (2 mL/plate). The sarkosyl was squirted on the plate two to three times to ensure complete and rapid lysis. Cell lysates were layered onto a cesium chloride gradient in polyallomer tubes. The CsCl density gradients contained four different density steps (1.72, 1.49, 1.02, 0.84 g/mL). To prepare the gradients, a stock solution of CsCl (density = 1.86 g/mL) was diluted with 1 × TE (10 mM Tris-HCl, pH 7.5, 1 mM EDTA). The tubes were centrifuged in a Beckman SW41 rotor at 125,000 × g for 20 h at 20 °C. Fractions (0.2 mL) were collected from the top of the gradients. The DNA concentration was determined using a microplate reader at 260 nm (EON, BioTek, USA).

All fractions were diluted with the equivalent volume of 25 mM sodium phosphate buffer (pH 6.5) for 15 min, and blotted on a nitrocellulose membrane. After soaking the membrane in TBST (10 mM Tris-HCl, pH 7.5, 150 mM NaCl) for 15 min and blocking with TBST containing 5% nonfat dried milk for 2 h, the complexes of hTop2β-DNA and free hTop2β were detected using specific antibody probe (Proteintech Group, USA) at a 1:10000 dilution. The IRDye 800CW Goat anti-rabbit (LI-COR Biosystems, USA) (1:15000) was used as secondary antibody. The fluorescence signals were analyzed using the Odyssey imaging system (LI-COR Biosystems, USA).

### DNA cleavage assay

Effect of compound C-Bi on hTop2β was measured using a topoisomerase II drug screening kit (TopoGEN, USA). The cleavage reactions were carried out in a final volume of 20 μL, containing 100 ng of supercoiled pHOT1 plasmid DNA, 3 units of hTop2β, 0–100 μM compound C-Bi (dissolved in 1% DMSO), and complete assay buffer (5×). After incubation at 37 °C for 30 min, the reaction was terminated by the addition of 2 μL sodium dodecyl sulphate (10%). Then the reaction mixtures were incubated with 2 μL of proteinase K (50 μg/mL) at 37 °C for 15 min to digest hTop2β. Final samples were mixed with 2 μL loading buffer (10×) and subjected to electrophoresis in 1% agarose.

### Surface plasmon resonance

The SPR assay was performed at 25 °C using a BI2000 SPR system (BioSensing Instrument, USA). Before determination, 1 × cysteamine hydrochloride (Sigma-Aldrich) was covered onto the sensor chip in a dark. After 12 h incubation, the chip was further covered by the mixture solution of dextran sodium salt, N-hydroxysuccinimide and N-(3-dimethylaminopropyl)-N′-ethylcarbodiimide hydrochloride (All from Sigma-Aldrich) for 3 h. hTop2β and DNA (in a 1.2-fold molar ratio to protein) were incubated at 4 °C for 1 h, and then the hTop2β-DNA binary complex was immobilized onto the sensor chip. The running buffer was PBS (pH 6.5) containing 5% DMSO. 1 M ethanolamine hydrochloride solution (30 μL/min, 100 μL) was then injected to block the unreacted ester group. After that, compound C-Bi was injected to flow across the chip surface with the concentrations from 50 to 800 μM at a flow rate of 60 μL/min. Each analyte was in triplicate determination. Between measurements, a regeneration cycle was completed by applying 20 mM NaOH over the sensor chip to dissociate the compound. The kinetic parameters provided estimates of both association and dissociation rate constants (*k*_*a*_ and *k*_*d*_), and from these values the equilibrium parameter (*K*_*D*_) for evaluating binding affinity of hTop2β-DNA for DMEP derivative was calculated using the relationship *K*_*D*_ = *k*_*d*_/*k*_*a*_.

### Crystallization

The protein sample was mixed with 2 mM compound C-Bi (in DMSO) and DNA substrate (in a 1.2-fold molar ratio to protein). Initial crystallization trials for the hTop2β^core^-DNA-compound C-Bi complex were performed as previously reported^20^. 1 μL of concentrated hTop2β^core^-DNA- compound C-Bi was mixed with an equal amount of reservoir solution and equilibrated against 500 μL of reservoir solution at 4 °C. The complex was crystallized by the hanging-drop vapor diffusion method using 100 mM magnesium chloride, 50 mM sodium citrate (pH 5.3), and 20% 2-methyl-2,4-pentanediol (MPD) as the precipitating agent, and 0.05% ethyl acetate (Hampton Research, USA) was used as additive. Crystals reached the maximum dimensions in around two weeks. Crystals were harvested by transferring into mother liquor containing 30% MPD and 1 mM corresponding compound before looping and flash-freezing in liquid nitrogen for data collection.

### Structure determination

The X-ray diffraction data on the hTop2β^core^-DNA complex were collected at the Shanghai Synchrotron Radiation Facility (SSRF) (Shanghai, China) beamline BL18U and BL19U1^30^ and were processed using the HKL3000 program suite. The structure was solved by molecular replacement with the PHENIX MR (using the structure of hTop2β^core^-DNA-VP-16 (PDB: 3QX3)^20^ as the search model). Then the structure was built and refined by Coot^31^ and PHENIX^32^ and analyzed by PyMOL^33^.

### Accession code

The crystal structure of the complex of hTop2β^core^-DNA, has been deposited with the RCSB Protein Data Bank under the accession code 5ZAD.

## Electronic supplementary material

Supplementary information
