# Supplementary material for: Increasing the distance between two monomers of topoisomerase IIβ under the action of antitumor agent 4β-sulfur-(benzimidazole) 4′-demethylepipodophyllotoxin
Source: Sci Rep. 2018 Oct 8;8:14949. doi: 10.1038/s41598-018-33366-2 (PMC6175940; doi:10.1038/s41598-018-33366-2)
Supplement: Supplementary file 1 — Supplementary information [file 41598_2018_33366_MOESM1_ESM.pdf]

Supplementary information

Increasing the distance between two monomers of topoisomerase IIβ under the action of antitumor agent 4*β*-sulfur-(benzimidazole) 4'-demethylepipodophyllotoxin

Lin-Yang Sun [1], Li-Wen Zhu [1], Ya-Jie Tang *

Hubei Key Laboratory of Industrial Microbiology, Hubei Provincial Cooperative Innovation Center of Industrial Fermentation, Key Laboratory of Fermentation Engineering (Ministry of Education), Hubei University of Technology, Wuhan 430068 China

[1] These authors contributed equally to this work.

* Corresponding author. Tel. & Fax: +86-27-59750491. Email: yajietang@QQ.com (Y-J Tang);

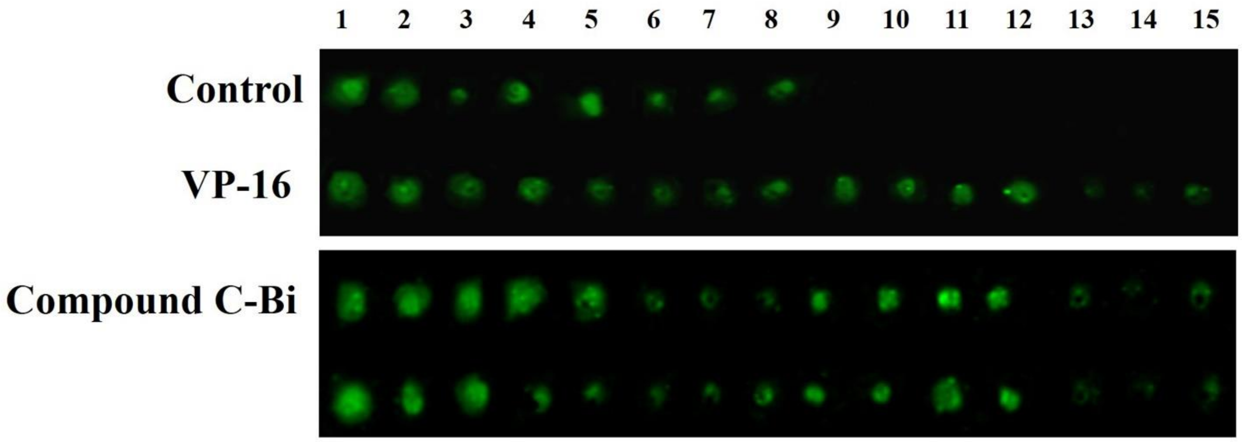

Figure S1. Full length blots of the cropped images of Figure 1B. Unlabeled lanes on the blot represent other conditions that are not relevant for this study.

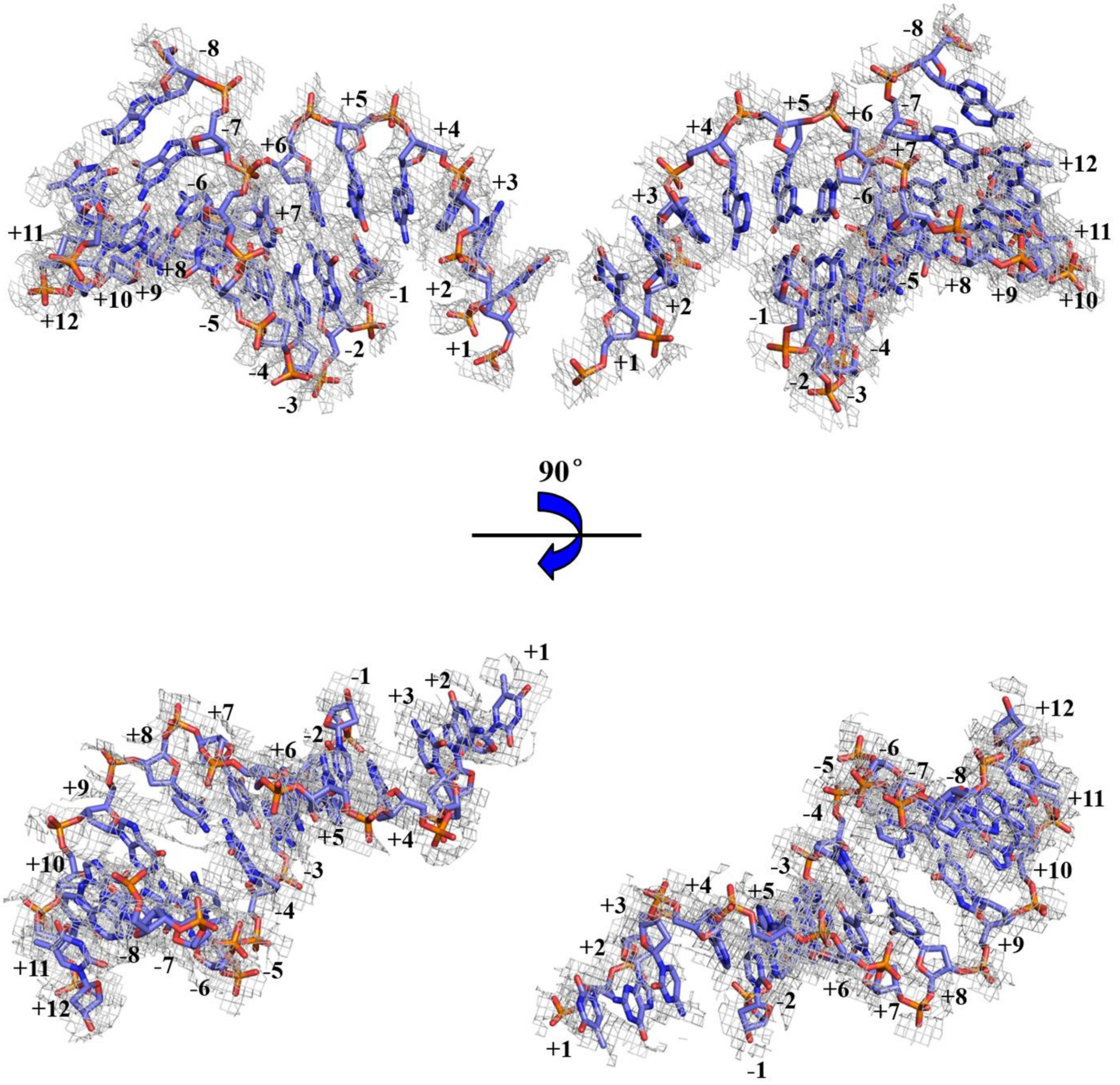

Figure S2. The electron density maps of the DNA in the hTOP2β^{core}-DNA binary cleavage complex.

Table S1. Kinetics parameters of the interactions of VP-16 and compound C-Bi to hTop2β-DNA complex.

	VP-16			Compound C-Bi		
k_a ($M^{-1}s^{-1}$)	64.21	62.89	59.73	343.8	373.81	349.3
k_d (s^{-1})	0.006851	0.007214	0.007132	0.015712	0.015887	0.017323
K_D (μM)	106.7	114.7	119.4	45.7	42.5	49.6

Table S2. Distances between selected groups in Top2-DNA complexes.

Structure	Distance (Å)				Reference
	Drug-binding site (R503)	Active site (Y821)	DNA-intercalating site (I872)	Cleavage site (3'-OH of the -1 nucleotide)	
5ZAD	34.54	16.26	58.71	38.48	This work
3QX3	26.62	19.84	52.65	31.70	1
4J3N	25.72	20.87	52.76	30.35	2
4G0U	24.11	23.76	50.99	27.17	2
3L4K	-	23.63	47.71	29.74	3

References

1. Wu, C. C. *et al*. Structural basis of type II topoisomerase inhibition by the anticancer drug etoposide. *Science* **333**, 459-462 (2011).
2. Wu, C. C., Li, Y. C., Wang, Y. R., Li, T. K. & Chan, N. L. On the structural basis and design guidelines for type II topoisomerase-targeting anticancer drugs. *Nucleic Acids Res*. **41**, 10630-10640 (2013).
3. Collins, T. R. L., Hammes, G. G. & Hsieh, T. S. Analysis of the eukaryotic topoisomerase II DNA gate: A single-molecule FRET and structural perspective. *Nucleic Acids Res*. **37**, 712-720 (2009).
